# 3,3′-(Piperazine-1,4-diium-1,4-di­yl)di­propionate dihydrate

**DOI:** 10.1107/S1600536812037312

**Published:** 2012-09-01

**Authors:** Shouwen Jin, Yanfei Huang, Hao Fang, Tianyi Wang, Liangliang Ding

**Affiliations:** a Tianmu College of ZheJiang A & F University, Lin’An 311300, People’s Republic of China

## Abstract

During the recrystallization of 3-[4-(2-carb­oxy­eth­yl)piperazin-1-yl]propionic acid, the carb­oxy­lic acid H atoms were transferred to the piperazine N atoms, forming the title compound, C_10_H_18_N_2_O_4_·2H_2_O, in which the zwitterion lies about an inversion center. In the crystal, bifurcated N—H⋯(O,O) hydrogen bonds connect the zwitterions into a two-dimensional framework parallel to (-102) forming *R*_4_^4^(30) rings. O—H⋯O hydrogen bonds involving the solvent water mol­ecules connect the two-dimensional framework into a three-dimensional network. In addition, weak C—H⋯O hydrogen bonds are observed.

## Related literature

For general background and applications of carb­oxy­lic acids, see: Jin *et al.* (2012 ▶); Grossel *et al.* (2006 ▶); Rueff *et al.* (2001 ▶); Strachan *et al.* (2007 ▶); Desiraju (2002 ▶). For hydrogen-bond motifs, see: Bernstein *et al.* (1995 ▶). 
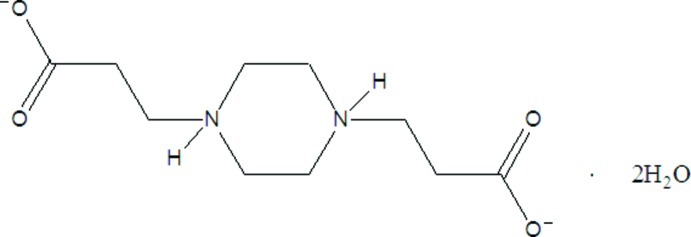


## Experimental

### 

#### Crystal data

C_10_H_18_N_2_O_4_·2H_2_O
*M* *_r_* = 266.30
Monoclinic, 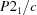

*a* = 6.8028 (6) Å
*b* = 8.8925 (7) Å
*c* = 10.4301 (11) Å
β = 101.780 (1)°
*V* = 617.67 (10) Å^3^
*Z* = 2
Mo *K*α radiation
μ = 0.12 mm^−1^
*T* = 298 K
0.43 × 0.40 × 0.32 mm

#### Data collection

Bruker SMART CCD diffractometer
Absorption correction: multi-scan (*SADABS*; Bruker, 2002 ▶) *T*_min_ = 0.951, *T*_max_ = 0.963
2951 measured reflections
1087 independent reflections
895 reflections with *I* > 2σ(*I*)
*R*_int_ = 0.023

#### Refinement

*R*[*F* ^2^ > 2σ(*F* ^2^)] = 0.040
*wR*(*F* ^2^) = 0.111
*S* = 1.06
1087 reflections
82 parameters
H-atom parameters constrained
Δρ_max_ = 0.21 e Å^−3^
Δρ_min_ = −0.23 e Å^−3^

### 

Data collection: *SMART* (Bruker, 2002 ▶); cell refinement: *SAINT* (Bruker, 2002 ▶); data reduction: *SAINT*; program(s) used to solve structure: *SHELXS97* (Sheldrick, 2008 ▶); program(s) used to refine structure: *SHELXL97* (Sheldrick, 2008 ▶); molecular graphics: *SHELXTL* (Sheldrick, 2008 ▶) and *Mercury* (Macrae *et al.*, 2006 ▶); software used to prepare material for publication: *SHELXTL*.

## Supplementary Material

Crystal structure: contains datablock(s) global, I. DOI: 10.1107/S1600536812037312/lh5520sup1.cif

Structure factors: contains datablock(s) I. DOI: 10.1107/S1600536812037312/lh5520Isup2.hkl

Supplementary material file. DOI: 10.1107/S1600536812037312/lh5520Isup3.cml

Additional supplementary materials:  crystallographic information; 3D view; checkCIF report

## Figures and Tables

**Table 1:** Hydrogen-bond geometry (Å, °)

*D*—H⋯*A*	*D*—H	H⋯*A*	*D*⋯*A*	*D*—H⋯*A*
O3—H3*F*⋯O2^i^	0.85	1.93	2.776 (2)	177
O3—H3*E*⋯O1	0.85	2.11	2.964 (2)	177
N1—H1⋯O2^ii^	0.91	2.50	3.0577 (19)	120
N1—H1⋯O1^ii^	0.91	1.80	2.7011 (18)	172
C4—H4*B*⋯O3^iii^	0.97	2.58	3.419 (2)	145
C4—H4*B*⋯O2^ii^	0.97	2.53	3.137 (2)	120
C5—H5*A*⋯O1^iv^	0.97	2.51	3.477 (2)	172

Symmetry codes: (i) 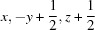; (ii) 
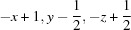
; (iii) 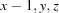; (iv) 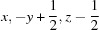.

**Fig. 1.**
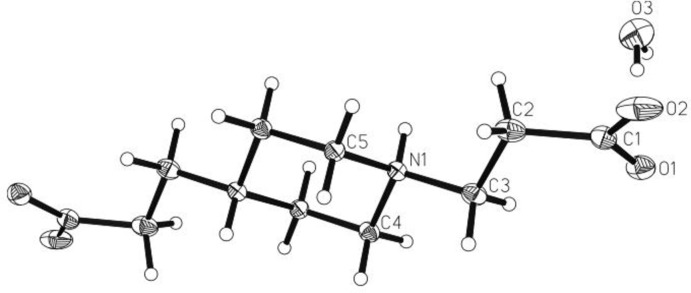
The molecular structure of (I) with displacement ellipsoids drawn at the 30% probability level. Unlabeled atoms are related by the symmetry operator (-x, -y, -z). Only the symmetry unique solvent water molecule is shown.

**Fig. 2.**
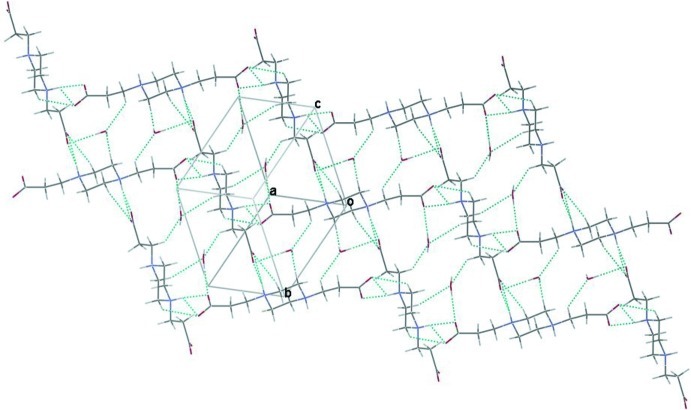
Part of the crystal structure with hydrogen bonds shown as dotted lines.

## supplementary crystallographic information

### Comment

Carboxylic acids are important compounds, which have been widely used in various fields as coordination chemistry (Rueff *et al.*, 2001), pharmaceutical chemistry (Strachan *et al.*, 2007), and supramolecular chemistry (Desiraju, 2002). Recently the main focus for carboxylic acids has been in crystal engineering *via* hydrogen bonded assembly of organic acids and organic bases (Grossel *et al.*, 2006). As an extension of our study concentrating on hydrogen bonded assembly of organic acids and organic bases (Jin *et al.*, 2012), herein we report the crystal structure of the title compound (I).

During the recrystallization of 3-[4-(2-carboxy-ethyl)-piperazin-1-yl]-propionic acid the carboxylic acid H atoms were transferred to the piperazine N atoms forming (I) (Fig. 1) in which the zwitterion lies across an inversion center. In the crystal, bifurcated N—H···(O,O) hydrogen bonds connect the zwitterions a two-dimensional framework parallel to (102) forming R^4^_4_(30) rings (Bernstein *et al.*, 1995). Furthermore O—H···O hydrogen bonds involving sovent water molecules connect the two-dimensional framework into a three-dimensional network. In addition, weak C—H···O hydrogen bonds are observed (Fig. 2).

### Experimental

3-[4-(2-Carboxy-ethyl)-piperazin-1-yl]-propionic acid (23.0 mg, 0.10 mmol) was dissolved in 6 ml of ethanol, and pyridine (15.8 mg, 0.2 mmol) was added to the ethanol solution. The solution was stirred for 1 h, and then filtered into a test tube. The solution was left standing at room temperature for about one week, colorless block crystals were obtained.

### Refinement

All H atoms were visible in difference Fourier maps. They were subsequently included in calculated positions with C—H = 0.97 Å, N—H = 0.91Å, O—H = 0.85Å and were constrained to ride on their parent atoms with *U*_iso_(H) = 1.2U_eq_(C,N,O).

### Crystal data

**Table:**

C_10_H_18_N_2_O_4_·2H_2_O	*F*(000) = 288
*M**_r_* = 266.30	*D*_x_ = 1.432 Mg m^−^^3^
Monoclinic, *P*2_1_/*c*	Mo *K*α radiation, λ = 0.71073 Å
Hall symbol: -P 2ybc	Cell parameters from 1525 reflections
*a* = 6.8028 (6) Å	θ = 3.0–28.2°
*b* = 8.8925 (7) Å	µ = 0.12 mm^−^^1^
*c* = 10.4301 (11) Å	*T* = 298 K
β = 101.780 (1)°	Block, colorless
*V* = 617.67 (10) Å^3^	0.43 × 0.40 × 0.32 mm
*Z* = 2	

### Data collection

**Table:**

Bruker SMART CCD diffractometer	1087 independent reflections
Radiation source: fine-focus sealed tube	895 reflections with *I* > 2σ(*I*)
Graphite monochromator	*R*_int_ = 0.023
φ and ω scans	θ_max_ = 25.0°, θ_min_ = 3.0°
Absorption correction: multi-scan (*SADABS*; Bruker, 2002)	*h* = −8→7
*T*_min_ = 0.951, *T*_max_ = 0.963	*k* = −10→5
2951 measured reflections	*l* = −12→11

### Refinement

**Table:**

Refinement on *F*^2^	Primary atom site location: structure-invariant direct methods
Least-squares matrix: full	Secondary atom site location: difference Fourier map
*R*[*F*^2^ > 2σ(*F*^2^)] = 0.040	Hydrogen site location: inferred from neighbouring sites
*wR*(*F*^2^) = 0.111	H-atom parameters constrained
*S* = 1.06	*w* = 1/[σ^2^(*F*_o_^2^) + (0.0526*P*)^2^ + 0.3063*P*] where *P* = (*F*_o_^2^ + 2*F*_c_^2^)/3
1087 reflections	(Δ/σ)_max_ < 0.001
82 parameters	Δρ_max_ = 0.21 e Å^−^^3^
0 restraints	Δρ_min_ = −0.23 e Å^−^^3^

### Special details

**Table:**

Geometry. All e.s.d.'s (except the e.s.d. in the dihedral angle between two l.s. planes) are estimated using the full covariance matrix. The cell e.s.d.'s are taken into account individually in the estimation of e.s.d.'s in distances, angles and torsion angles; correlations between e.s.d.'s in cell parameters are only used when they are defined by crystal symmetry. An approximate (isotropic) treatment of cell e.s.d.'s is used for estimating e.s.d.'s involving l.s. planes.
Refinement. Refinement of *F*^2^ against ALL reflections. The weighted *R*-factor *wR* and goodness of fit *S* are based on *F*^2^, conventional *R*-factors *R* are based on *F*, with *F* set to zero for negative *F*^2^. The threshold expression of *F*^2^ > σ(*F*^2^) is used only for calculating *R*-factors(gt) *etc*. and is not relevant to the choice of reflections for refinement. *R*-factors based on *F*^2^ are statistically about twice as large as those based on *F*, and *R*- factors based on ALL data will be even larger.

### Fractional atomic coordinates and isotropic or equivalent isotropic displacement parameters (Å^2^)

**Table:**

	*x*	*y*	*z*	*U*_iso_*/*U*_eq_	
N1	0.15979 (19)	0.08898 (15)	0.08004 (12)	0.0204 (3)	
H1	0.2428	0.0121	0.1137	0.024*	
O1	0.56844 (18)	0.37957 (15)	0.31249 (12)	0.0340 (4)	
O2	0.7046 (2)	0.4517 (2)	0.14744 (14)	0.0551 (5)	
O3	0.8070 (2)	0.10838 (18)	0.40824 (15)	0.0541 (5)	
H3E	0.7405	0.1861	0.3781	0.065*	
H3F	0.7788	0.0870	0.4818	0.065*	
C1	0.5838 (2)	0.3752 (2)	0.19346 (17)	0.0288 (4)	
C2	0.4531 (3)	0.2636 (2)	0.10320 (18)	0.0335 (5)	
H2A	0.4302	0.3019	0.0143	0.040*	
H2B	0.5256	0.1694	0.1049	0.040*	
C3	0.2521 (3)	0.23274 (19)	0.13874 (17)	0.0276 (4)	
H3A	0.2697	0.2274	0.2333	0.033*	
H3B	0.1616	0.3154	0.1084	0.033*	
C4	−0.0379 (2)	0.06441 (19)	0.11862 (16)	0.0236 (4)	
H4A	−0.1283	0.1462	0.0851	0.028*	
H4B	−0.0187	0.0648	0.2134	0.028*	
C5	0.1314 (2)	0.08320 (19)	−0.06607 (15)	0.0230 (4)	
H5A	0.2604	0.0950	−0.0910	0.028*	
H5B	0.0456	0.1656	−0.1042	0.028*	

### Atomic displacement parameters (Å^2^)

**Table:**

	*U*^11^	*U*^22^	*U*^33^	*U*^12^	*U*^13^	*U*^23^
N1	0.0183 (7)	0.0214 (7)	0.0211 (7)	−0.0005 (5)	0.0031 (5)	−0.0011 (6)
O1	0.0355 (7)	0.0386 (8)	0.0274 (7)	−0.0108 (6)	0.0049 (5)	−0.0046 (6)
O2	0.0584 (9)	0.0713 (11)	0.0388 (8)	−0.0414 (9)	0.0174 (7)	−0.0142 (8)
O3	0.0650 (10)	0.0500 (10)	0.0518 (10)	0.0019 (8)	0.0225 (8)	0.0053 (8)
C1	0.0261 (9)	0.0297 (9)	0.0300 (10)	−0.0040 (7)	0.0044 (7)	−0.0036 (8)
C2	0.0317 (10)	0.0379 (11)	0.0317 (10)	−0.0112 (8)	0.0084 (8)	−0.0090 (8)
C3	0.0249 (9)	0.0256 (9)	0.0317 (9)	−0.0040 (7)	0.0046 (7)	−0.0064 (7)
C4	0.0201 (8)	0.0287 (9)	0.0224 (8)	0.0000 (7)	0.0056 (6)	−0.0013 (7)
C5	0.0214 (8)	0.0279 (9)	0.0202 (8)	−0.0010 (7)	0.0050 (6)	0.0022 (7)

### Geometric parameters (Å, º)

**Table:**

N1—C4	1.4966 (19)	C2—H2A	0.9700
N1—C5	1.4975 (19)	C2—H2B	0.9700
N1—C3	1.499 (2)	C3—H3A	0.9700
N1—H1	0.9100	C3—H3B	0.9700
O1—C1	1.267 (2)	C4—C5^i^	1.512 (2)
O2—C1	1.237 (2)	C4—H4A	0.9700
O3—H3E	0.8501	C4—H4B	0.9700
O3—H3F	0.8500	C5—C4^i^	1.512 (2)
C1—C2	1.523 (2)	C5—H5A	0.9700
C2—C3	1.513 (2)	C5—H5B	0.9700
			
C4—N1—C5	109.42 (12)	N1—C3—H3A	109.2
C4—N1—C3	109.84 (12)	C2—C3—H3A	109.2
C5—N1—C3	113.65 (13)	N1—C3—H3B	109.2
C4—N1—H1	107.9	C2—C3—H3B	109.2
C5—N1—H1	107.9	H3A—C3—H3B	107.9
C3—N1—H1	107.9	N1—C4—C5^i^	111.35 (13)
H3E—O3—H3F	108.3	N1—C4—H4A	109.4
O2—C1—O1	123.88 (16)	C5^i^—C4—H4A	109.4
O2—C1—C2	117.96 (16)	N1—C4—H4B	109.4
O1—C1—C2	118.09 (15)	C5^i^—C4—H4B	109.4
C3—C2—C1	114.16 (15)	H4A—C4—H4B	108.0
C3—C2—H2A	108.7	N1—C5—C4^i^	110.85 (13)
C1—C2—H2A	108.7	N1—C5—H5A	109.5
C3—C2—H2B	108.7	C4^i^—C5—H5A	109.5
C1—C2—H2B	108.7	N1—C5—H5B	109.5
H2A—C2—H2B	107.6	C4^i^—C5—H5B	109.5
N1—C3—C2	112.25 (14)	H5A—C5—H5B	108.1
			
O2—C1—C2—C3	−151.50 (18)	C5—N1—C4—C5^i^	57.11 (18)
O1—C1—C2—C3	31.5 (2)	C3—N1—C4—C5^i^	−177.48 (13)
C4—N1—C3—C2	179.91 (14)	C4—N1—C5—C4^i^	−56.82 (18)
C5—N1—C3—C2	−57.14 (19)	C3—N1—C5—C4^i^	−179.99 (13)
C1—C2—C3—N1	−160.56 (15)		

Symmetry code: (i) −*x*, −*y*, −*z*.

### Hydrogen-bond geometry (Å, º)

**Table:**

*D*—H···*A*	*D*—H	H···*A*	*D*···*A*	*D*—H···*A*
O3—H3*F*···O2^ii^	0.85	1.93	2.776 (2)	177
O3—H3*E*···O1	0.85	2.11	2.964 (2)	177
N1—H1···O2^iii^	0.91	2.50	3.0577 (19)	120
N1—H1···O1^iii^	0.91	1.80	2.7011 (18)	172
C4—H4*B*···O3^iv^	0.97	2.58	3.419 (2)	145
C4—H4*B*···O2^iii^	0.97	2.53	3.137 (2)	120
C5—H5*A*···O1^v^	0.97	2.51	3.477 (2)	172

Symmetry codes: (ii) *x*, −*y*+1/2, *z*+1/2; (iii) −*x*+1, *y*−1/2, −*z*+1/2; (iv) *x*−1, *y*, *z*; (v) *x*, −*y*+1/2, *z*−1/2.
